# Gut bacteria require neutrophils to promote mammary tumorigenesis

**DOI:** 10.18632/oncotarget.3328

**Published:** 2015-03-20

**Authors:** Jessica R. Lakritz, Theofilos Poutahidis, Sheyla Mirabal, Bernard J. Varian, Tatiana Levkovich, Yassin M. Ibrahim, Jerrold M. Ward, Ellen C. Teng, Brett Fisher, Nicola Parry, Stephanie Lesage, Natalie Alberg, Sravya Gourishetti, James G. Fox, Zhongming Ge, Susan E. Erdman

**Affiliations:** ^1^ Division of Comparative Medicine, Massachusetts Institute of Technology, 77 Massachusetts Avenue, Cambridge, MA 02139, USA; ^2^ Laboratory of Pathology, Faculty of Veterinary Medicine, Aristotle University of Thessaloniki, Greece 54124; ^3^ Global VetPathology, Montgomery Village, MD 20886, USA

**Keywords:** enteric, bacteria, microbes, mammary cancer, immune system

## Abstract

Recent studies suggest that gastrointestinal tract microbiota modulate cancer development in distant non-intestinal tissues. Here we tested mechanistic hypotheses using a targeted pathogenic gut microbial infection animal model with a predilection to breast cancer. FVB-Tg(C3-1-TAg)cJeg/JegJ female mice were infected by gastric gavage with *Helicobacter hepaticus* at three-months-of-age putting them at increased risk for mammary tumor development. Tumorigenesis was multifocal and characterized by extensive infiltrates of myeloperoxidase-positive neutrophils otherwise implicated in cancer progression in humans and animal models. To test whether neutrophils were important in etiopathogenesis in this bacteria-triggered model system, we next systemically depleted mice of neutrophils using thrice weekly intraperitoneal injections with anti-Ly-6G antibody. We found that antibody depletion entirely inhibited tumor development in this *H. hepaticus*-infected model. These data demonstrate that host neutrophil-associated immune responses to intestinal tract microbes significantly impact cancer progression in distal tissues such as mammary glands, and identify gut microbes as novel targets for extra-intestinal cancer therapy.

## INTRODUCTION

Breast cancer is a leading cause of neoplasia-associated mortality [1]. While studying intestinal cancer in mouse models, we observed that targeted orogastric infection with *Helicobacter hepaticus*, a murine bacterium frequently colonizing the gut of mice [2–4], causes mammary neoplasia with particularly increased frequency [5]. We hypothesized that unbalanced host immune responses to enteric bacteria may promote the development of cancer in epithelia distant from the gut [6]. Since that time, genetically-engineered and diet-associated mouse models of cancer, wound healing, and obesity have provided evidence to support our original hypothesis that gut bacteria have consequences in systemic immunity [7–9].

Several lines of evidence support the roles of GI tract microbial flora in promoting intestinal neoplasmatogenesis [3, 5, 10–14]. However, linking gut bacteria with systemic innate immune-related effects that enhance tumor formation throughout the body expands this paradigm [5, 6]. This notion is challenging, especially in the light of recent reports demonstrating that gut microbe dysbiosis undermines the outcome of both immune and non-immune chemotherapeutic cancer treatment modalities [15, 16]. In order to identify the key immune cell players, prior studies have used combinations of immunohistochemistry, targeted immune depletions, and adoptive cell transfers [5, 17–19]. In those studies, reciprocal relationships exist between innate immune neutrophils [11] and anti-inflammatory Interleukin (IL)-10-dependent activities of CD4+ T regulatory (Treg) lymphocytes that bestow immune homeostasis [5, 10, 20]. Indeed, neutrophils have been identified as important contributors of cancer initiation and development [21]. Through the generation of reactive oxygen species (ROS), neutrophils induce genotoxic damage, thus contributing to the mutagenic events in the initial steps of carcinogenesis [22–24]. Also, cytokines and chemokines as well as serine proteases secreted by tumor-associated neutrophils shape the tumor microenvironment and promote tumor growth [25, 26].

In the present study, we used female genetically-engineered FVB-Tg(C3-1-TAg)cJeg/JegJ mice with a predilection to develop mammary cancer [27]. In this model system, the C3(1)/SV40 T antigen is over-expressed after exposure to sex steroid hormones during development of prostate and mammary cancer [28, 29]. This mouse model recapitulates all stages of human mammary carcinomas. These lesions have been previously classified as infiltrating ductal carcinomas, including ductal epithelial cell atypia and mammary intraepithelial neoplasia (MIN), which corresponds to the human ductal carcinoma *in situ* [29]. Here we record that targeted orogastric infection with *H. hepaticus* increases mammary tumor multiplicity recapitulating the classical C3-1-TAg mouse mammary tumorigenesis pattern. Further, systemic depletion of neutrophils, a key innate immune inflammatory cell, can block this extra-intestinal tumorigenic phenomenon. These data demonstrate that host inflammatory responses to environmental microbes significantly impact cancer progression in distant non-intestinal tissues by a neutrophil-mediated mechanism.

## RESULTS

### Orogastric gavage with *Helicobacter hepaticus* increases mammary tumor burden in genetically-prone C3-1-TAg mice

**Figure 1:**
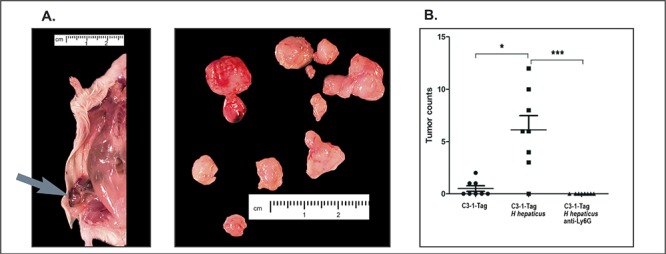
Tumor multiplicity assessment in experimental groups of 15-week-old C3-1-TAg mice **(A)** Grossly visible tumors (arrow) found in each mouse were removed, placed on a petri dish and counted. **(B)** The statistical analysis of mammary tumor count data shows that infection with *H. hepaticus* accelerates tumorigenesis, whereas the depletion of neutrophils negates this effect. The y-axis depicts the mean ± SEM of mammary tumor counts. *p < 0.05. The points correspond to the mean of total tumors counted in each mouse.

It was previously shown that infection with enteropathogenic *H. hepaticus* rapidly induced mammary tumor formation in genetically-susceptible Apc^Min/+^ [ApcMin] mice [5, 6, 30, 31]. However, the use of ApcMin mice as a model of mammary cancer has certain peculiarities, raising doubts about broader relevancy of roles of gut microbiota in mammary epithelial carcinogenesis. To examine this evident gut microbe-mammary linkage further, we first tested orogastric challenge with *H. hepaticus* in the FVB-Tg(C3-1-TAg)cJeg/JegJ mouse model [29]. Within three weeks of infection, we found numerous small palpable tumors arising in multiple mammary tissue sites of three-month-old C3-1-TAg mice infected with *H. hepaticus* (Figure 1A). By comparison, sham media-dosed matched control animals had significantly fewer palpable tumors (Figure 1B).

**Figure 2:**
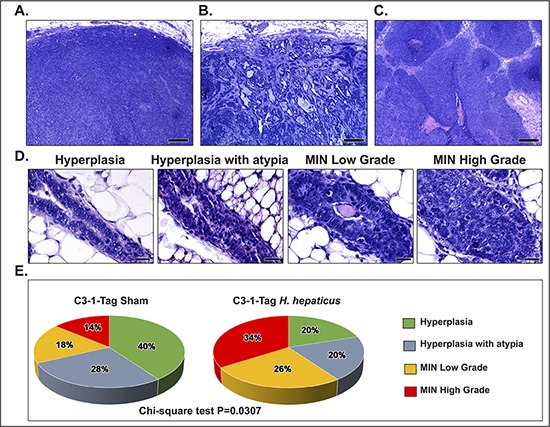
Effects of *H. hepaticus* on mammary gland carcinogenesis Tumors of both *H. hepaticus*-infected and uninfected control C3-1-TAg mice shared similar histomorphological patterns. **(A)** Neoplastic cells arranged in solid sheets, cords or nests with minimal gland formation and small amounts of intervening stroma. **(B)** Glandular-like growth was seen in occasional areas at the periphery of the tumors. Note irregular glands in moderate to large amounts of desmoplastic stroma. **(C)** Large solid cord arrangement of neoplastic cells with variably sized areas of either diffuse or commedo type intratumoral necrosis. **(D)** The initial stages of mammary tumorigenesis. From left to the right there is progressively increased epithelial pseudostratification, cellular atypia, nuclear pleomorphism and mitotic figures. **(E)** Classification of abnormal glands in non-tumoral areas according to their histological stage. The mammary glands of *H. hepaticus*-infected mice are in more advanced stages of neoplastic progression compared to those of uninfected controls. Hematoxylin and Eosin (A, B, C and D); Scale bars: 250 μm (A, B and C) and 25 μm (D).

The unencapsulated expansile tumors in both *H. hepaticus*- and sham-treated mice had the typical C3-1-TAg mouse mammary adenocarcinoma histomorphology (Figures 2A–2C). In the non-tumoral mammary tissue, ducts and terminal duct lobular units (TDLU) showed a spectrum of hyperplastic, preneoplastic, and early neoplastic lesions depicting the well-characterized stages of the C3-1-TAg mouse mammary tumorigenesis progression (Figure 2D). In the non-tumoral areas the affected mammary epithelia of *H. hepaticus*-treated mice appeared to be in more advanced tumorigenesis stages by comparison with the uninfected controls. In order to confirm this observation we determined histomorphometrically the percentage of abnormal glands at each one of four critical histological stages of mammary tumorigenesis in *H. hepaticus*-infected and non-infected mice. We found that the classification of abnormal glands according to their histological stage differed significantly between experimental groups (*P* = 0.0307), with *H. hepaticus*-infected mice having a higher percentage of abnormal glands with mammary intraepithelial neoplasia (MIN) when compared to controls (Figure 2E).

### Gut microbial challenge leads to up-regulation of inflammatory cells in mammary tissue

**Figure 3:**
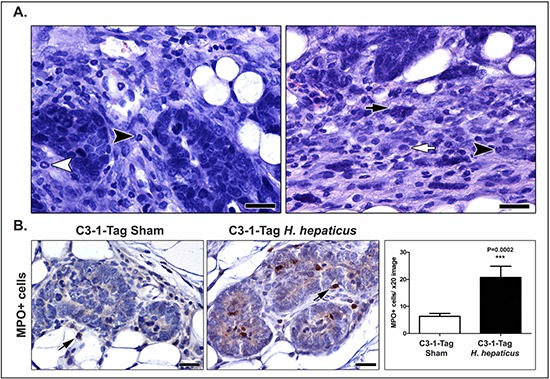
*H. hepaticus* infection up-regulates MIN-associated neutrophils **(A)** The tumor-associated inflammation was comparable in large-sized neoplasms of both *H. hepaticus*-infected and uninfected control mice. Neutrophils (black arrow-heads), myeloid precursor cells (white arrow-head), mast cells (black arrow) and macrophages (white arrow) at the periphery of tumors are shown. **(B)** Morphometric counts of MPO-positive cells (arrows) in MIN lesions. The numbers of neutrophils are significantly higher in *H-hepaticus* infected mice compared to controls. Hematoxylin and Eosin (A) IHC; Diaminobenzidine chromogen, Hematoxylin counterstain (B) Scale bars: 25 μm (A) and 50 μm (B) Numbers on the y-axis of bar graph correspond to the mean ± SEM of MPO+cells. ***p < 0.0001.

Knowing that inflammatory cells and factors were pivotal in etiopathogenesis of microbe-induced mammary [5, 17, 32] and prostate [18] tumors, we next examined whether inflammatory cells were increased in C3-1-TAg mice undergoing infection with *H. hepaticus*. We found the tumor-associated inflammatory cell component residing at the periphery of well-defined tumors (Figure 3A) consisted of abundant macrophages, neutrophils, myeloid precursor cells with ring-shaped nuclei [33, 34], mononuclear cells, and mast cells. The same types of inflammatory cells were found in the connective tissue stroma within the tumor, with the exception of mast cells, which were sparse. Mast cells as well as neutrophils, however, were topographically associated with early neoplastic lesions such as MIN in the non-tumoral areas of the mammary glands.

Comparing histomorphologically similar MIN lesions in the two experimental groups, we noticed that neutrophils accumulated in higher numbers around the MIN lesions when mice were infected with *H. hepaticus*. Tumor associated neutrophils (TAN) were previously shown to enhance tumorigenesis in mouse models [35]. To quantify this result we performed counts of myeloperoxidase (MPO)-positive cells (neutrophils) in histologically comparable MIN lesions of both *H. hepaticus*-infected and uninfected mice. We found that the *H. hepaticus* infection status correlated with higher numbers of MIN-associated MPO-positive cells in statistically significant levels (*P* = 0.002) (Figure 3B).

### Systemic depletion of neutrophils inhibits mammary tumor formation

**Figure 4:**
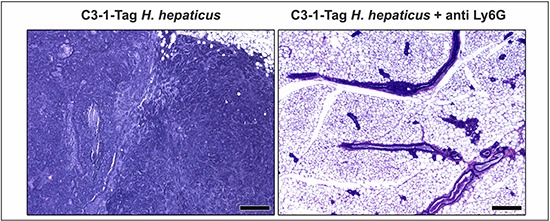
The depletion of neutrophils with anti-Ly-6G antibody blocks effect of *H. hepaticus*-promoted C3-1-TAg mouse mammary carcinogenesis Fifteen-weeks-old *H. hepaticus*-infected C3-1-TAg mice had the typical for this mouse model mammary tumors. At the same timepoint their anti-Ly6G-treated counterparts had early neoplastic changes but no tumors at all. Hematoxylin and Eosin. Scale bars: 250 μm

Finally, based upon earlier findings showing that neutrophils are a consistent feature of *H. hepaticus*-induced inflammation [36] and cancer [10, 11, 20], and due to the emerging role of neutrophils in carcinogenesis [21, 37, 38], we tested whether Ly-6G+ neutrophils were required for mammary cancer in this mouse model. To test this we used thrice-weekly intraperitoneal injections with anti-Ly6G clone 18A previously shown to target mature neutrophils in mice [39]. While *H. hepaticus*-infected mice had multifocal well-sized mammary adenocarcinoma tumors (8/9, 88%), *H. hepaticus*-infected mice that were treated with anti-Ly6G antibody had no evidence of mammary adenocarcinoma tumors (0/9, 0%) (Figure 1B). The neutrophil depleted mice had only preneoplastic and early neoplastic lesions in their mammary gland epithelia (Figure 4). Specifically, the most advanced histopathological lesion found [7] was low and high grade MIN (carcinoma *in situ*) in three mice (3/9, 33%), hyperplasia with marked atypia in two (2/9, 22%), hyperplasia with atypia, intermediate grade in one (1/9, 0.09%) hyperplasia without atypia, low grade in three mice (3/9, 33%).

## DISCUSSION

In the present study we use the well-characterized C3-1-TAg mouse model of mammary cancer to show that bacteria residing in the gut mucosa affect the evolution of neoplastic lesions in mammary glands. We find here that the neutrophil is an important cellular element of this mechanism, which links innate immune and neoplastic events occurring in topographically distant epithelia. This highlights the neutrophil as an important mediator of the remote effect of gut microbiota on mammary epithelial carcinogenesis. Further, these findings build upon the pivotal role of systemic immune balance in emerging pre-neoplastic lesions throughout the body not obviously linked with chronic inflammation.

Previously we have shown that *H. hepaticus*-infected C57BL/6 ApcMin Rag2-deficient female mice develop mammary tumors with increased frequency [5]. This was the first study providing compelling experimental evidence linking the effects of gastrointestinal bacteria with mammary carcinogenesis. Further, this provided evidence that innate immunity alone was sufficient for distant carcinogenesis triggered by gut bacterial infection. Subsequently we showed that male ApcMin mice infected with *H. hepaticus* develop prostate tumors transplantable to uninfected mice using purified lymph node cells obtained from *H. hepaticus*-infected mice alone [18], implicating systemic innate immune cell trafficking in distant carcinogenic events. Indeed, the abundance of morphologically-distinctive myeloid cells throughout target tissues in the ApcMin model supports the notion of gut bacteria-triggered systemic trafficking of immune precursor cells [18]. However, the use of ApcMin mice as a model of mammary or prostate carcinogenesis raises some questions. These mice exhibit a predilection for intestinal polypoid adenomas, due to their heterogygous *adenomatosis polyposis coli* gene status [30] and accelerated thymic involution [40]. Given microbe-dependent intestinal polyposis in this model [5, 41], it is difficult to assess whether the mammary cancer is due to *H. hepaticus* infection *per se* or to microbe-increased multiplicity of intestinal polyps. Taken together, these facts raise doubts about roles of gut microbiota in mammary epithelial carcinogenesis beyond ApcMin mice. The results of the present paper contribute towards showing that *H. hepaticus* accelerates mammary carcinogenesis in other animal systems, in this case the C3-1-TAg female mouse that is a widely utilized mouse model for hormonally-dependent cancer [28]. Thus the present findings build upon our previous observations and expand upon the ApcMin mouse mammary cancer paradigm.

Further, these data provide additional evidence that breast cancer is associated with microbial dysbiosis in the gut. Altering gut microbes can regulate the immune system and lower the risk of breast cancer; in particular, overgrowth or lack of certain types of bacteria in the gut have been associated with many diseases ranging from obesity, to digestive disorders, to cancers [42]. Differences in the bacterial populations in breast tumor tissue and healthy breast tissue have been reported [43]. Frequent use of antibiotics that may disrupt the microbiome is associated with breast cancer development and relapse [44–46]. Interestingly, immune dysregulation can be transferred in mice by fecal microbe transplant [47–50]. Additionally, the microbiome plays a key role in estrogen cycling in the body, and gut dysbiosis results in higher circulating estrogens which has been linked to postmenopausal breast cancer [51–54]. Estrogen and neutrophil dysregulation are hallmarks of breast cancer development [55], and a high neutrophil to lymphocyte ratio is associated with breast cancer relapse in patients. Both these cell populations are clearly modulated by the microbiome and inflammation [56–59].

Interestingly, our current results suggest that the remote effect of gut microbiota in mammary carcinogenesis does not specifically depend on the molecular pathway of mammary carcinogenesis involved. C3-1-TAg mice develop mammary cancer due to hormonally-dependent functional inactivation of the P53 and Rb tumor suppressor genes, which leads mammary epithelia to cell cycle regulation defects, uncontrolled proliferation and resistance to apoptosis [29]. The inactivation of these tumor suppressors and the dysregulation of the cell growth and apoptosis pathways they control are common in human breast cancer [29, 60]. Although the role of the *APC* gene, which is central in the β-catenin/WNT pathway of carcinogenesis in human breast cancer is emerging [61], neither *APC* mutant mice nor humans carrying germline mutations in *APC* develop spontaneously mammary cancer in a high percentage [62, 63]. By contrast C3-1-TAg mice inevitably develop mammary neoplasms [29]. This fact presumably reflects the more universal role of the P53 and Rb genes in the various types of neoplastic disease compared to the APC gene.

Our compiled results also show that the effect of intestinal *H. hepaticus* on mammary carcinogenesis does not connect specifically with the histological type of the mammary tumor. Previously we found *H. hepaticus* promotes formation of mammary adenosquamous carcinoma in ApcMin mice [5]. Here, we find in the C3-1-TAg mouse that it accelerates the growth of solid carcinomas, resembling infiltrating ductal carcinomas of humans [29, 64, 65].

Importantly, the C3-1-TAg mice on a FVB background develop neither IBD nor intestinal tumors [29]. This suggests that subclinical, rather than overt, alterations in the immune status are sufficient for the induction of phenotypically distinct effects on mammary carcinogenesis. Other proliferative and neoplastic pathologies reported to arise in different anatomical sites of female C3-1-TAg mice, such as the sweat glands of the foot pads, the salivary glands, and the vomeronasal organ [29] were not observed in the present study. This lack of tumors in other sites was predictable since these pathologies occur in older animals more than five-months-of-age, a time point that was not reached with young (less than four-months-of-age) mice in our present study. Unlike ApcMin, the C3-1-TAg mice are immunocompetent, which suggests that immune system defects are not necessary for the remote effect of GI tract bacteria on mammary carcinogenesis. Although T regulatory (Treg) cells function reciprocally with neutrophils [36], the roles for Treg cells in preventing mammary neoplasia were not specifically examined in this study. Knowing that C3-1-TAg mice exhibit hormone-dependent carcinogenesis [29], and that neutrophils robustly interact with estrogen and IL-10 [66], the relationships between microbes, hormones, immunity and cancer are a key topic for future studies.

It appears that *H. hepaticus* is not unique among gut bacteria exerting effects on mammary gland tissue. Our other recent findings show that the probiotic bacterium *Lactobacillus reuteri*, when introduced in the GI tract flora of mice, has the opposite effect of suppressing mammary tumor formation in genetically-susceptible HER2 mutant mice [7]. In both cases involving gut microbiota, whether the acceleration of mammary carcinogenesis by pathogenic *H. hepaticus* or suppression of mammary tumorigenesis by probiotic *L. reuteri*, we discover differences in the immune cell composition of the mammary gland microenvironment. An interesting aspect is that these gut-mammary connections may be covert; even in the absence of demonstrable inflammatory disease in the gut or elsewhere, we find that *H. hepaticus*-infected mice have not only higher risk for cancer but also more frequent inflammatory cells associating with mammary glands at early neoplastic stages.

In the present study we found that neutrophils associate with mammary lesions in higher numbers in the *H. hepaticus*-infected mice when compared to their uninfected controls. Neutrophils are a consistent feature of *H. hepaticus*-induced inflammation [36] and cancer [10, 11, 20]. Other evidence for the role of neutrophils in carcinogenesis and tumor evolution is emerging and the therapeutic approach of targeting the tumor-associated neutrophils has been recently introduced [21]. Studies in samples from many different types of human tumors suggest that a high neutrophil to lymphocyte ratios predicts a poor clinical outcome [38]. Also, the depletion of neutrophils has been shown to block cancer evolution in mouse models of fibrosarcoma [67], pancreatic islet cell [68], colonic [11], and pulmonary carcinoma [35].

Taken together with our earlier data these facts led us to test whether the depletion of neutrophils could affect mammary carcinogenesis in this mouse model, as well. Recognizing the etiopathogenic potential of myeloid immune precursor trafficking in lymph nodes, spleen, and target tissues after *H. hepaticus* infection [18], it's possible that depleting Ly-6G+ cells includes other relevant immune cells; although, clone 1A8 was previously shown to be specific for mature neutrophils [39]. We found that the *H. hepaticus*-infected C3-1-TAg mice depleted of Ly-6G+ neutrophils had significantly fewer mammary lesions, and in those cases only preneoplastic and early neoplastic lesions, in contrast to their non-depleted matching controls which exhibited palpable mammary tumors. The dependence of mammary tumorigenesis upon the presence of neutrophils highlights the neutrophil as an important mediator of the remote effect of gut microbiota on mammary epithelial carcinogenesis. Adoptive transfer experiments [18] using highly purified gut bacteria-stimulated myeloid and neutrophil cell populations will address this in future studies.

In conclusion, the results of the present study indicate that gut microbiota bacterial elements, in the absence of overt inflammatory disease, contribute to the character of the subclinical, systemic inflammatory tone of the mammalian organism. Based on these findings it appears that systemic inflammatory tone, which is mediated, at least in part, by neutrophils, affects the evolution of preneoplastic lesions to cancer in epithelia locating distantly from the gut, such as those of the mammary glands.

## METHODS

### Animals

Genetically-inbred FVB-Tg(C3-1-TAg)cJeg/JegJ female mice (Jackson Labs, Bar Harbor, ME), were housed and handled in Association for Assessment and Accreditation of Laboratory Animal Care (AAALAC)-accredited facilities with diets, experimental methods, and housing as specifically approved by the Institutional Animal Care and Use Committee. Experimental design was to expose mice to *Helicobacter hepaticus* infection by gastric gavage at the age of twelve weeks, and then monitor for three weeks until euthanasia using carbon dioxide overdose. Control mice of the same age underwent gastric gavage with media alone. Subsets of infected mice were injected intraperitoneally with anti-Ly-6G antibody to deplete neutrophils, or with sham isotype-matched antibody alone, thrice weekly for 3.5–4 weeks starting three days before infection. Each experimental group included 5–10 animals per group with one replicate experiment with statistically similar outcomes to validate results. Mammary tissues were collected upon necropsy and then examined histologically.

### Experimental infection with *Helicobacter hepaticus*

A total of 51 experimental mice were dosed at 12 weeks of age with *H. hepaticus* and then housed separately in a bio-containment area within the same animal facility. *H. hepaticus* (strain 3B1, ATCC #51449) [2] was grown under microaerobic conditions, prepared, and confirmed to be pure as described elsewhere [3]. Experimental mice received 0.2 ml of fresh inoculum by gastric gavage every other day for a total of three doses. Cecum and stool were collected at necropsy 3–4 weeks post-infection and analyzed by PCR using *H. hepaticus*-specific primers to confirm bowel colonization.

### Systemic depletion of neutrophils

Mice were treated with anti-Ly6G antibody (clone 1A8; Bio-X-Cell, West Lebanon, NH) at 200 ug per mouse intraperitoneally 3X weekly for 3–4 weeks starting 3 days before infection with *H. hepaticus*. Treatment with antibody continued concurrent with *H. hepaticus* infection for a duration of 3 weeks, with euthanasia occurring at age 15 weeks. Treated mice were then compared with mice receiving a comparable dose of sham isotype antibody alone. Depletion of neutrophils was confirmed by undetectably low levels of MPO+ cells in spleens of mice treated with anti-Ly-6G antibody compared to sham-treated controls.

### Statistical analyses

The Mann-Whitney *U* test was used for analyzing histomorphometry data. Mammary tumor multiplicity was evaluated using the Kruskal-Wallis analysis followed by the Dunn's multiple comparison test. Mammary gland lesion staging comparison between groups was done with the Chi-square test. Replicate experiments were not significantly different. A *p*-value < 0.05 was statistically significant.

### Histopathology and immunohistochemistry

For histologic evaluation, formalin-fixed tissues were embedded in paraffin, cut at 5 μm, and stained with hematoxylin and eosin. Hyperplastic and preneoplastic lesions in the mammary gland were staged based on previously published consensus criteria [65] as previously described [7]. Rabbit polyclonal antibodies against Myeloperoxidase (MPO) (ThermoFisher Scientific/Lab Vision, Fremont, CA), were used for immunohistochemistry. Heat-induced antigen retrieval was performed with citrate buffer, pH 6. Rabbit primary antibody binding was detected with goat anti-rabbit polymer HRP (ZytoChem Plus, Berlin, Germany). Color was developed with DAB substrate-chromogen system (ThermoFisher Scientific/Lab Vision) and tissues were counterstained with hematoxylin.

For quantitative histomorphometry, MPO-positive cells were counted in 20 randomly-selected images of x20 representative high power fields using the ImageJ processing and analysis program (NIH, Bethesda, MD) as previously described [7] and results were recorded as number of cells per image. The relative percentage of abnormal glands belonging to different histological stages of mammary tumorigenesis in *H. hepaticus*-infected and non-infected mice was determined in 50 abnormal mammary duct profiles from each group. For that, histological sections from non-tumoral areas of 5 randomly selected mice per experimental group were used. Starting from the upper left corner of each histological section and moving towards its lower right, the first 10 abnormal glands (hyperplastic or with early neoplasia) found were staged and the result was recorded.
